# Heart Rate and Distance Measurement of Two Multisport Activity Trackers and a Cellphone App in Different Sports: A Cross-Sectional Validation and Comparison Field Study

**DOI:** 10.3390/s22010180

**Published:** 2021-12-28

**Authors:** Mario Budig, Michael Keiner, Riccardo Stoohs, Meike Hoffmeister, Volker Höltke

**Affiliations:** 1 Department of Sports Science, German University of Health & Sport, 85737 Ismaning, Germany; Mario.Budig@edu.my-campus-berlin.com (M.B.); Meike.Hoffmeister@dhgs-hochschule.de (M.H.); Volker.hoeltke@dhgs-hochschule.de (V.H.); 2 Somnolab, 44263 Dortmund, Germany; stoohs@somnolab.de

**Keywords:** wearables, sport app, accuracy, validity, field study

## Abstract

Options for monitoring sports have been continuously developed by using activity trackers to determine almost all vital and movement parameters. The aim of this study was to validate heart rate and distance measurements of two activity trackers (Polar Ignite; Garmin Forerunner 945) and a cellphone app (Polar Beat app using iPhone 7 as a hardware platform) in a cross-sectional field study. Thirty-six moderate endurance-trained adults (20 males/16 females) completed a test battery consisting of walking and running 3 km, a 1.6 km interval run (standard 400 m outdoor stadium), 3 km forest run (outdoor), 500/1000 m swim and 4.3/31.5 km cycling tests. Heart rate was recorded via a Polar H10 chest strap and distance was controlled via a map, 400 m stadium or 50 m pool. For all tests except swimming, strong correlation values of r > 0.90 were calculated with moderate exercise intensity and a mean absolute percentage error of 2.85%. During the interval run, several significant deviations (*p* < 0.049) were observed. The swim disciplines showed significant differences (*p* < 0.001), with the 500 m test having a mean absolute percentage error of 8.61%, and the 1000 m test of 55.32%. In most tests, significant deviations (*p* < 0.001) were calculated for distance measurement. However, a maximum mean absolute percentage error of 4.74% and small mean absolute error based on the total route lengths were calculated. This study showed that the accuracy of heart rate measurements could be rated as good, except for rapid changing heart rate during interval training and swimming. Distance measurement differences were rated as non-relevant in practice for use in sports.

## 1. Introduction

Wrist-worn activity trackers and wearables are currently very popular among the active general population as well as among competitive athletes. By 2019, given direct Internet cloud connection, there were 722 million connected wearables worldwide, which provide an unprecedented amount of data for science [1] and commerce. The measured data include not only sports-related data but also data on common everyday activities such as climbing stairs, steps, walking or hiking, and other parameters such as sleep or breathing at night [2] and heart rate variability (HRV) [3]. This enormous potential is of particular interest in the field of sports, especially in the collection of heart rate (HR)- and distance-related data based on photoplethysmography (PPG) and global navigation satellite system (GNSS)/GPS sensors in activity trackers [4]. In recent years, numerous new validation studies of HR measurements as well as of the number of daily steps and distance have been carried out with a laboratory testing focus. The study by Boudreaux, et al. [5] showed very accurate HR values during cycling and resistance training (r > 0.79) with a mean absolute percentage error (MAPE) = 6.24% with Apple Watch Series 2. Tedesco, et al. [6] examined during a treadmill test series in distance measurement an r < 0.50 and MAPE up to 123.76%, and in HR measurement an r > 0.70 with MAPE up to 13.89%. Chow and Yang [7] reported for resting HR and in series with moderate physical activity high correlation values (r > 0.80) with MAPE < 10% for young and older adults. Some researchers have combined laboratory and field studies. The study by Höchsmann, et al. [8] investigated walking and showed a very small MAPE of < 3% for treadmill and a higher MAPE up to 47% for free-living tests. Müller, et al. [9] showed for both laboratory and free-living tests with moderate physical activity moderate-to-high correlations of r > 0.51–0.83 with MAPE up to 13% for HR measurement. The study by Düking, et al. [10] postulated similar results, with r = 0.54–0.99 in a validation study of four activity trackers including vigorous activity. Henriksen, et al. [11] showed with Polar Vantage V and Oura Ring a correlation of r > 0.75 with MAPE 72%, with resting HR r = 0.90 with MAPE 3% in a free-living setting. Today, pure field studies are underrepresented, especially for the discipline of swimming. The studies by Xie, et al. [12], Budig, et al. [13] and Navalta, et al. [14] showed strong correlation values with constantly and smoothly changing HR during moderate training loads (r = 0.96) and low-to-moderate correlation with rapidly changing HR between r = 0.31–0.58 in running, trail running, and cycling. Düking, et al. [10] documented pure correlations for vigorous activity including a shuttle run test for Garmin Fenix 5 and Fitbit Versa, and good correlations for Polar Vantage V and Apple Watch Series 4. Navalta, et al. [14] calculated an MAPE up to 24% in their trail running study. In swimming, Olstad [15], Olstad, et al. [16] and Olstad and Zinner [17] investigated HR measurement and obtained a moderate correlation of r = 0.59. For distance measurement, most studies showed significant differences of *p* < 0.001. However, low MAE and MAPE values based on total track length could be calculated in general [12,13,18,19]. Benson, et al. [18] showed mostly an underestimation in speed and distance with different GPS mobile apps using an iPhone. Gilgen-Ammann, et al. [19] investigated walking, running and cycling under various outdoor conditions and showed small MAPE up to 6.1% with mostly underestimation of distance as well.

The aim of the present study was to determine the accuracy of the HR and distance measurements of two multisport activity trackers and a cellphone app in different sports conditions such as walking, running, cycling, and especially swimming, with various activity intensities and lengths in pure field tests.

## 2. Materials and Methods

### 2.1. Participants

Thirty-six (20 males/16 females) moderately endurance-trained participants (mean age: 36.1 ± 12.8; mean height: 176.1 ± 9.6 cm; mean weight: 73.3 ± 14.4 kg; mean body mass index: 23.4 ± 2.7 kg/m^2^; mean activity level of 5.1 ± 1.2) participated voluntarily in the field tests. To fairly represent everyday and general use, the subjects were only restricted by age (18–65 years) and activity level (5–8) in accordance with previous research [20,21,22,23]. Prior to each test, the subjects provided information about their health status using the PAR-Q questionnaire [24].

All participants provided written informed consent to participate in the present study. Approval for this study was obtained from the institutional review board and ethics committee of the German University of Health & Sport, reference number: 08/2019.1. The study was performed with human participants in accordance with the Helsinki Declaration.

### 2.2. Design and Procedures

The study was designed as a cross-sectional field study based on the study by Budig et al. [24]. For a differentiated investigation of distances and different heart rate ranges in potential training scenarios, the sports running, swimming and cycling were chosen, with different lengths and differences in altitude and speeds. The subjects completed a standardized test protocol on 3 separate test days. During all tests, heart rate and distance measurements were recorded by two activity trackers, a chest strap and a cellphone app. Immediately after each test, the resting HR was additionally recorded over a six-minute period with the exception of walking and swimming. On each testing day, a 10 min standardized sport-specific functional exercise warm-up was performed [25] to provide enough individual adaptation time to the optical sensor system [26] and to prepare each participant for the exercise.

On testing day 1, the test battery (performed in a 400 m outdoor stadium) included 3 km of walking with an individual walking speed sufficient to cause an increased HR. After a 3 min break, the participants ran 3 km at a moderate running speed (sufficient to lead to a more elevated HR compared to walking). After a break of 15 min, an interval run test of 1.6 km was performed. The interval run test included 400 m of trotting and 100 m with increased running speed until the individual’s maximum running speed was reached, followed by 100 m of trotting, 100 m of increased running speed, 100 m of trotting, 200 m of running at a submaximal speed (approximately 80% of the individual’s maximum running speed), 200 m of trotting, 200 m of running at a submaximal speed, and 200 m of trotting. The intervals were executed in one single sequence without a break.

On testing day 2 (5 to 7 days later) the running and cycling tests were executed on predetermined routes. Firstly, a forest run of 3.1 km in length and a geographical difference of ± 110 height in meters (hm) at moderate running speed were performed. Secondly, after a break of 30 min the participants biked (mountain bike) two times for 4.34 km (off-road), initially at a speed of 20–25 km per hour (km/h) and about +82/−69 hm. Thirdly, they performed a speed bike run of about 31.5 km, with a faster pace of 28–30 km/h and ±270 hm on normal roads.

On testing day 3, 5–7 days later, the swimming tests were executed in a 50 m Olympic pool at a speed of 2:00–2:15 min per 100 m, starting with a total length of 500 m followed by a short break and a total length of 1000 m. To ensure that all participants could cover the entire swimming distance with one swimming style, the breaststroke was chosen.

Both activity trackers, Garmin Forerunner 945 (Garmin Ltd., 2019; Olathe, KS, USA, firmware 5.50) [26] and Polar Ignite (Polar Electro Oy, 2019; Kempele, Finland, firmware 2.0.25) [27], were randomly fitted tightly, alternately on the left and right forearm behind the processus styloideus ulnae. The chest strap Polar H10 (Polar Electro Oy, 2018; Kempele, Finland, Firmware 3.0.56) [28] was used to determine the criterion measurement of HR. The validity of the Polar chest strap was confirmed in previous studies [3,9,16,29]. The cellphone app Polar Beat (Polar Electro Oy, 2020; Kempele, Finland, version 3.4.7) running with an iPhone 7 (Apple Inc., 2017, Cupertino, CA, USA, firmware 13.6) was used to control the chest strap and as a third distance measurement. The phone was alternately held in the left and right hands during the tests. All devices recorded at 1 s intervals and were started and stopped by the test leader in a standardized sequence. Start: 1. Polar Chest Strap via the Polar Beat app; 2. Garmin 945; 3. Polar Ignite; and Stop: vice versa. Polar Ignite timing was used as the reference timing. All test data were saved and stored on each device separately. For the criterion measurement of distance, the software MagicMaps3D (MagicMaps GmbH, Reutlingen, Germany, version 1.5.0) using an official map set from the Federal State Survey Office of North Rhine-Westphalia, Germany [30], and distance calculations of 400 m in the stadium and 50 m in the Olympic swimming pool were used.

### 2.3. Statistical Analysis

For all devices, as soon as the test was stopped and the tracker/chest strap had established a connection via Bluetooth with the cellphone apps Garmin Connect (Garmin Ltd., Olathe, KS, USA, version: 4.37.2.0) and Polar Flow (Polar Electro Oy, Kempele, Finland, version 4.8.0), the measurement data were automatically transferred to the test leaders Garmin/Polar cloud accounts. The respective test data were extracted from the cloud via Polar Flow and Garmin Connect software on the computer in the TCX data format (Training Center Extensible Markup Language). Golden Cheetah software (Cranleigh, UK, version 3.5) was used for further data extraction. The HR and distance data were exported to and summarized with MS Excel 2016 (Microsoft, Redmond, WA, USA). To make sure all trackers and chest strap were running with the same timing, all devices were connected to the computer or cellphone app once a day using the network time protocol (NTP), which continuously synchronizes to the atomic time of the Physikalisch-Technische Bundesanstalt (Physikalisch-Technische Bundesanstalt, Braunschweig, Germany). The Polar Ignite was used to standardize the start/stop sequence. The tracker was the last to start and the first to stop in order to get the reference timing for each test for subsequent synchronization of all devices.

All statistical analyses were performed using Microsoft Excel 2016 and IBM SPSS (version 24.0, Armonk, NY, USA). The significance level was set at *p* < 0.05. Descriptive data analyses of each subject’s physical data were performed, and the normal distribution of all data was assessed using the Kolmogorov–Smirnov test. After all *t*-test analyses, false discovery rate (FDR) corrections were performed to exclude experimental errors [31]. The following statistical tests were based on the recommendations for wearable validation and assessment selected by Düking, Fuss, Holmberg and Sperlich [20], and Nelson, Low, Jacobson, Areán, Torous and Allen [21]. They were also used in other previous studies [3,32,33]. Correlations of the HR measurements were calculated between the Garmin and Polar results, and the criterion measurement (two-way random, absolute agreement) was considered high >0.79, moderate 0.40–0.79 or low <0.40. Difference analyses for HR and distance measurements between the criterion measurements and the Garmin, Polar Ignite and Polar Beat app (using the iPhone GPS data) results were calculated using the MAE and mean absolute percentage error (MAPE) [|(mean difference activity tracker − criterion measurement)/mean criterion measurement| × 100]. The level of agreement (LoA) was calculated for the HR measurement between the Garmin and Polar Ignite results and the criterion measurement. LoA was assessed as described by Bland–Altman and was expressed using a Bland–Altman plot [34]. T-tests were calculated for all HR and for distance tests between the Garmin 945, Polar Ignite, cellphone app and for the criterion measurement followed by FDR correction and the effect size calculation according to Cohen’s d. T-tests were calculated for HRs with subinterval differentiation during the interval run test at every start, middle and end position/time of each subinterval, followed by FDR correction and effect size calculation. One-way ANOVA followed by Scheffé post hoc test was used to analyze the 6 min resting HR between all the activity trackers and chest strap measurements and between both activity trackers. Boxplot diagram analysis was used for the distance measurement between the Garmin 945, Polar Ignite, cellphone app and criterion measurement.

## 3. Results

**Figure 1:**
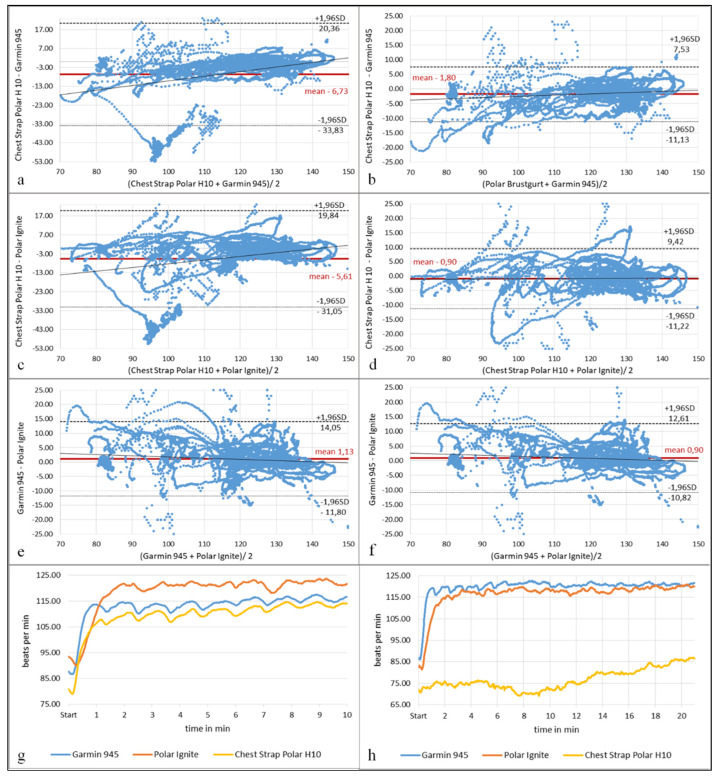
Bland–Altman diagrams of heart rate measurements (*n* = 36). (**a**) Garmin 945 vs. criterion, (**b**) Garmin 945 vs. criterion without swim, (**c**) Polar Ignite vs. criterion, (**d**) Polar Ignite vs. criterion without swim, (**e**) Garmin 945 vs. Polar Ignite, (**f**) Garmin 945 vs. Polar Ignite without swim, (**g**,**h**) heart rate lines Garmin 945, Polar Ignite and chest trap Polar H10 (**g**) swim 500 m, (**h**) swim 1000 m; min = minute, mean = mean value, SD = standard deviation of the difference.

**Figure 2:**
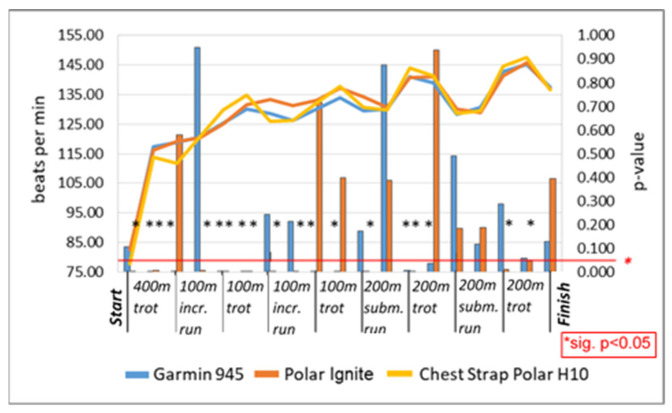
HR interval, 1.6 km interval run, *p*-value expressed in bars and corresponding HR graph at start/middle/end of each interval phase (*n* = 36); min = minute, incr. = increasing run, subm. = submaximal run, trot = trotting run; * = *p* < 0.05.

HR measurements showed strong correlations, with r = 0.90–0.92 (*p* < 0.001) (*n* = 344,960) between each activity tracker and the Polar chest strap H10 with the exception of swimming. The correlation coefficient including swimming was r = 0.71–0.88 (*p* < 0.001) (*n* = 418,323). The difference analysis of all HRs showed a small MAE of 3.29 bpm/MAPE of 2.85% without the swimming tests, and an MAE of 7.96 bpm/MAPE of 6.80% including swimming tests. The individual disciplines showed a maximum MAE of 6.76 bpm and MAPE of 5.92%, with the exception of the 500 m swim test, which exhibited an MAE of up to 9.45 bpm/MAPE of 8.61% and the 1000 m swim test, with an MAE of up to 42.72 bpm/MAPE of 55.32% (Table 1). The results of the Bland–Altman diagrams document the fluctuation range of differences with and without the swimming tests (Figure 1). In particular, the LoA differences between the diagrams showed deviations of up to 22.70 bpm for Garmin 945 and 19.83 bpm for Polar Ignite. The outlined HR graph in Figure 1h confirms this for the 1000 m swim test. The subinterval differentiation statistics for the 1.6 km interval run showed significant differences from the criterion measurement: eight for Garmin 945 and eleven for Polar Ignite out of 19 measurement points (start/middle/end of each subinterval phase). FDR adjusted up to *p* < 0.049 with effect sizes between d = 0.2 and 0.5. Eight tests were nonsignificant (*p* = 0.080–0.960). The HR graphs underline the significant differences (Figure 2). The *t*-test analyses of the other disciplines showed significant results only for the 3.1 km forest run, and both swim disciplines with *p* < 0.030 and d > 0.43 up to 2.34.

**Figure 3:**
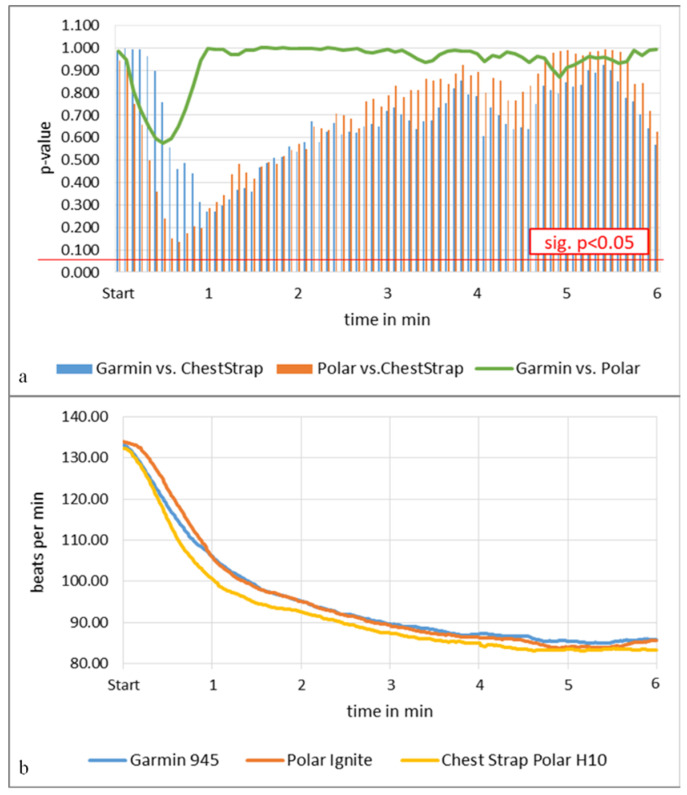
Resting heart rate (*n* = 36). (**a**) *p*-values expressed in bars, tracker compared to criterion measurement chest strap and as a line between both activity trackers, (**b**) corresponding HR graph, both trackers and the chest strap, min = minutes.

The resting HR ANOVA analyses of all tests except the swimming and walking tests showed no significant differences between both activity trackers and the chest strap or between activity trackers, expressed in Figure 3a. The only noticeable deviation was visible from the start of the measurement up to minute 1:30 with up to an 11 beats per minute (bpm) difference for both activity trackers, expressed in lines in Figure 3b. Polar Ignite showed slightly greater deviations than Garmin 945 (Figure 3). The correlation for both activity trackers was high between r = 0.83 and 0.96 (*p* < 0.001). The Bland–Altman plots confirmed these observations.

**Figure 4:**
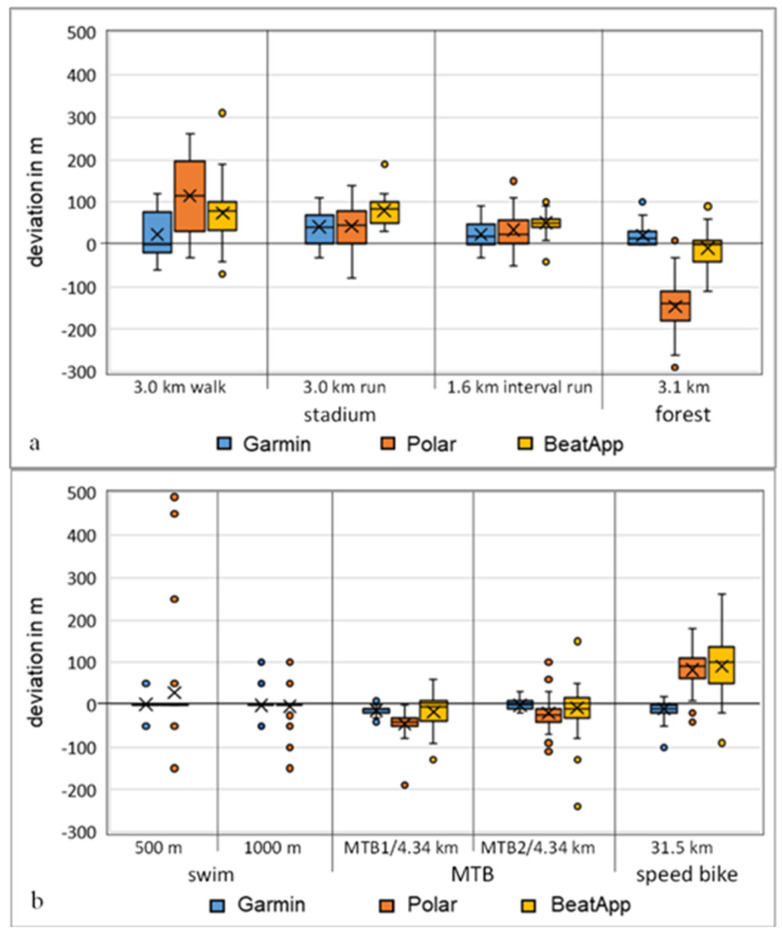
Boxplot of distance deviations from the criterion measurement in the various disciplines (*n* = 36). (**a**) *p*-values compared to criterion and between activity trackers, (**b**) corresponding heart rate graphs, min = minutes.

The *t*-test analyses of the two activity trackers and the iPhone app showed several significant results. The majority of differences in the running disciplines were highly significant for all measuring systems (*p* < 0.001). The cycling disciplines were significantly different for the Polar Ignite measurements (*p* < 0.002; d = 2.57). Garmin and the cellphone app showed no significant differences in cycling. The swimming results were unremarkable for all measurements. In further analyses of the mean error, no significant deviations were found despite statistically significant *t*-test results. The MAPE did not exceed 4.67% in any discipline. However, some very large maximum deviations were noticeable, such as swimming 500 m with +500 m and walking 3 km with +390 m. The largest deviations were observed with the Polar Ignite and the cellphone app, with mostly larger measurements. Garmin 945 had very small deviations in all measurements. All MAE/MAPE results are shown in Table 2 and illustrated in the boxplot in Figure 4.

## 4. Discussion

The aim of the study was to assess and validate the accuracy of two multisport activity trackers and a cellphone app for HR and distance measurements. The main findings support very accurate HR measurement data, including resting HR with moderately changing HR. However, with rapidly changing HR significant deviations with a measurement delay occurred. Significant HR differences were observed specifically in swimming conditions over 500 and 1000 m. For distance measurements, the calculated deviation was statistically significant, but with low MAE and MAPE the deviations were negligible for practical use.

For the HR measurements, we obtained similar high correlation values to those reported in previous studies [7,9,13,35,36] with the exception of those for swimming tests and the 1.6 km interval run. The resting HR measurement showed similar high correlation results to other studies as well [10,11,37], with the exception of Budig, et al. [13]. The error analysis showed similar clear statements as the correlation analysis. The MAEs were between 1 and 6 bpm with a minimum MAPE of 1.02% for speed bike cycling and a maximum MAPE of 5.92% for MTB2 cycling. The MAPE showed mostly a maximum of 3.0%. The exceptions were again the swimming disciplines with an MAE > 39.44 bpm and an MAPE up to 55.32%. When considering the level of error, the evaluation of each individual discipline that results in higher HR is significant. The shorter and more intense the cardiac load is, the higher the calculated error value and the resulting MAPE value, as in the MTB discipline. This finding is in line with the studies by Xie, et al. [12], Boudreaux, et al. [5], Müller, et al. [9] and Düking, et al. [10]. The Bland–Altman analysis showed consistent results in the assessment of LoA. Compared with the current studies, only Müller, et al. [9], Düking, et al. [10] and Navalta, et al. [14] showed slightly worse error values in a “free living” environment, in shuttle run and trail run disciplines with MAPE up to 24% when examining several wearables. The *t*-test analyses of the mean values have a limited significance since the number of measured values for the individual disciplines varied too much due to differences in the individual cycling and running speeds.

Additional *t*-test analyses of the subinterval phases at the start, middle and end of each subinterval of the 1.6 km interval run showed eight and eleven highly significant differences (*p* < 0.001; d > 0.3) out of nineteen measurement points for Polar Ignite and Garmin 945, respectively. The significant deviations were calculated during the 100 m increase runs between the 500 m and 700 m points and the submaximal run at the 1000 m point for both activity trackers, Polar Ignite was slightly different from than Garmin 945. A time delay in HR display could be observed for both activity trackers. The studies by Reddy, et al. [35] with high interval phases and Budig, et al. [13] with an identical test setup showed similar results. The studies by Düking, et al. [10] and Navalta, et al. [14] showed similar results in shuttle running and trail running tests.

The HR measurements with respect to the swimming discipline have to be discussed in more detail due to larger deviations. To date, the validation of wearables has been very poorly investigated, and only Olstad [15], Olstad, et al. [16] and Olstad and Zinner [17] have published data in this context. The 1000 m swimming measurements showed a highly significant difference (*p* < 0.001; d < −2.17) from the chest strap. A closer analysis reveals an MAE up to 42.72 bpm and an MAPE of up to 55.32% for both activity trackers. The examination of the HR graph clearly showed a significant measurement error of the chest strap Polar H10 with a permanently lower HR value (Figure 1h). In contrast, both activity trackers showed similar HR progression curves with an MAE of 3.28 bpm and an MAPE of 2.74%. Consequently, the chest strap Polar H10 could not be used as a valid reference measurement in the 1000 m swimming discipline. The 500 m measurement also showed significant differences (*p* < 0.001; d < −1.11), an MAE of 9.45 bpm, an MAPE of 8.61%, and correlation r = 0.08 for Polar Ignite and an MAE of 3.61 and an MAPE of 3.29% for Garmin 945, r = 0.49. This is in contrast to the studies by Olstad [15], Olstad, et al. [16] and Olstad and Zinner [17]. However, in these studies, the chest strap Polar H10 was worn under a triathlon swimsuit instead of uncovered on the skin. This study aimed to mimic general use of the chest strap, according to the chest strap manufacturer’s recommendations for usage [28]. Therefore, the wearing of the chest strap Polar H10 without a triathlon swimsuit could lead to major incorrect electrical measurements, depending on positioning and connection of the chest strap with the skin of the torso. The surrounded water could influence the electrical HR-conduction measurement due to its physical properties. The Bland–Altman analysis underlined this difference again. Overall, the 1000 m measurement cannot be used as a comparison measurement due to the permanent lower measurements of the Polar H10 chest strap. Therefore, the 500 m result must also be analyzed with caution. No validation study without the use of a swimsuit has yet been published.

For distance measurement, the *t*-test analysis showed that approximately half of all global positioning system (GPS)-based measurements had significant differences (*p* < 0.001 and d > 0.8). The stadium and forest 3.1 km runs were noticeable for all devices. For the other disciplines, Polar Ignite was significantly conspicuous overall, except in the swim disciplines. Garmin 945 and the Beat app were inconspicuous here. To date, there are still very few current studies examining GPS-based measurements in various disciplines [6,8,12,13,19]. Other studies have examined the use of external GPS receivers in combination with mobile apps in team sports [38,39,40]. In a further analysis, the majority of the running tests showed overestimation of distance measurement in all devices, with the largest deviations for the Polar Ignite measurements followed by Beat app and the smallest with Garmin 945. When considering the MAE, a decrease in the error level with increasing route length could be confirmed in absolute numbers. The Polar Ignite measurements for walking 3 km with an MAE of 117.22 m and for the speed bike of 31.5 km with an MAE of 85.56 m showed a very clear decrease. The values of the other disciplines behaved similarly, albeit to a lesser extent. This finding is in line with the studies by Xie, Wen, Liang, Jia, Gao and Lei [12] and Budig, Höltke and Keiner [13]. A possible influence of the routing due to the disturbance variables in connection with the global navigation satellite system (GNSS) measurement, such as poor reception of the satellite signals in the forest or in between high buildings in cities, could lead to additional measurements. The study of Gilgen-Ammann, et al. [19] concluded the same.

Both activity trackers use accelerometers as the primary data source for the swim and step count disciplines. Similar to GNSS studies, very few studies have been published concerning swim evaluation, but more for step counts in the last few years [41,42,43,44]. The distance measurements of the 500/1000 m swims showed no significant difference from the criterion. Garmin 945 showed lower values for MAE and MAPE than Polar Ignite. In particular, the high deviations of max +500 m and min −150 m for the 500 m swim test of the Polar Ignite were noticeable. A swimmer with poor swimming technique swam the large deviation. Due to the accelerometer measuring method, uncontrolled arm movements could lead to higher error values because the activity tracker accumulates the change in direction and the number of arm strokes per lane. The studies of Mooney, et al. [41] and Cossell [43] confirm this.

A limitation of this study is that only two activity trackers and one cellphone app were included. Therefore, a general statement about all wearables and apps is not possible. In principle, a test–retest procedure is recommended for a field test study design since the objectivity and reliability of tests that are difficult to standardize, such as forest runs or cycling, can be increased. Furthermore, the reference systems used can be validated again. As documented in this study, the chest strap Polar H10 measurement in the 1000 m swim was not valid. The only studies that validated chest strap Polar H10 in water used the strap under swim suits and are currently insufficient [16,17]. For further differentiation of GNSS-based distance measurements, measurements with and without routes in dense forests and deep valleys between high buildings in the city or tunnels and underpasses should be used in parallel. However, the route selection should be standardized for easy reproduction, such as 400 m stadium laps or staked bike routes, to reduce error possibilities.

## 5. Conclusions

This study confirms accurate HR measurement data obtained from common multisport activity trackers, including resting HR under conditions of moderately changing HR. This does not include all measuring conditions, as there were significant measurement differences in the swimming disciplines and significant deviations when using the chest strap without a swimsuit under water. Further clear limitations are the significant measurement inaccuracies in the case of rapidly changing HR during intensive interval training, as shown in the 1.6 km interval run test. The usage of the chest strap Polar H10 as a reference during in water tests has to be investigated further.

As for GNSS- and accelerometer-based distance measurements, the differences for all devices were mostly significant but not substantial enough for practical relevance due to the very low MAE/MAPE values. In isolated cases, the maximum deviation based on accelerometer measurement was very high. Due to the physical principles and the way of counting, wrist-worn activity trackers can lead to greater distance deviations due to poor swimming technique.

This study calculated similar results in the context of the existing studies [10,12,13,19,41,43]. HR and distance measurement accuracy can be described as sufficiently good for usage up to and including competitive sports, with the restrictions mentioned. However, follow-up studies are recommended with the newest devices, especially for swim tests, including the chest strap.

## Figures and Tables

**Table 1:** Heart rate measurement deviation calculation, mean absolute error, mean absolute percentage error (*n* = 36).

**HR Data**		**MAE**	**MAPE %**	**Min**	**Max**	**SD +/−**
HR all data (w/o swim)	Garmin vs. Chest Strap H10	3.29	2.85	0.00	21.25	3.89
	Polar Ignite vs. Chest Strap H10	3.23	2.80	0.00	23.36	4.25
	Garmin vs. Polar Ignite	3.23	2.75	0.00	19.58	5.11
Resting HR all data	Garmin vs. Chest Strap H10	2.71	2.93	0.43	5.62	1.14
	Polar Ignite vs. Chest Strap H10	2.69	2.91	0.35	8.48	1.96
	Garmin vs. Polar Ignite	1.03	1.09	0.00	4.80	1.12
HR interval 1.6 km	Garmin vs. Chest Strap H10	1.58	1.23	0.00	5.44	1.25
	Polar Ignite vs. Chest Strap H10	3.32	2.57	0.00	8.48	2.17
	Garmin vs. Polar Ignite	3.69	2.88	0.00	8.28	2.01
**Swim**		**MAE**	**MAPE** %	**Min**	**Max**	**SD +/−**
HR all data (with swim)	Garmin vs. Chest Strap H10	7.96	7.18	0.00	52.85	13.16
	Polar Ignite vs. Chest Strap H10	7.54	6.80	0.00	50.48	11.96
	Garmin vs. Polar Ignite	3.76	3.19	0.00	44.00	5.54
HR swim 500 m	Garmin vs. Chest Strap H10	3.61	3.29	1.56	9.75	1.59
	Polar Ignite vs. Chest Strap H10	9.45	8.61	0.11	14.56	2.67
	Garmin vs. Polar Ignite	6.79	5.99	0.06	12.53	1.83
HR swim 1000 m	Garmin vs. Chest Strap H10	42.72	55.32	14.31	52.85	6.04
	Polar Ignite vs. Chest Strap H10	39.44	51.08	9.07	50.48	6.71
	Garmin vs. Polar Ignite	3.28	2.74	0.03	20.81	3.07

MAE = mean absolute error (in beats per minute, absolute values), MAPE = mean absolute percentage error, min = minimum, max = maximum, SD = standard deviation, HR = heart rate, w/o = without, m = meter, km = kilometer.

**Table 2:** Distance measurement deviation calculation, mean absolute error, mean absolute percentage error (*n* = 36).

Discipline	Activity Tracker vs. Criterion Measurement	MAE	MAPE %	Min	Max	SD +/−
All data	Garmin 945	21.27	0.81	−60.00	120.00	25.80
	Polar Ignite	66.06	2.40	−30.00	500.00	58.88
	Beat App	59.29	1.67	0.00	310.00	48.93
3 km walk stadium	Garmin 945	46.39	1.55	−60.00	120.00	52.99
	Polar Ignite	117.22	3.91	−30.00	260.00	88.46
	Beat App	80.28	2.68	−70.00	310.00	67.46
3 km run stadium	Garmin 945	43.06	1.44	−30.00	110.00	38.18
	Polar Ignite	56.39	1.88	−80.00	140.00	53.50
	Beat App	80.00	2.67	30.00	190.00	31.08
1.6 km interval run stadium	Garmin 945	26.39	1.65	−30.00	90.00	30.16
	Polar Ignite	42.50	2.66	−50.00	150.00	44.49
	Beat App	53.61	3.35	−40.00	100.00	24.16
3.1 km forest run	Garmin 945	21.67	0.69	0.00	100.00	23.60
	Polar Ignite	146.67	4.67	−290.00	10.00	62.62
	Beat App	32.22	1.03	−110.00	90.00	43.67
Swim 500 m	Garmin 945	4.17	0.42	−50.00	50.00	14.57
	Polar Ignite	45.83	4.58	−150.00	500.00	122.11
Swim 1000 m	Garmin 945	9.72	0.97	−50.00	100.00	25.32
	Polar Ignite	17.36	1.74	−150.00	100.00	39.71
MTB1 4.34 km	Garmin 945	13.61	0.31	−40.00	10.00	10.91
	Polar Ignite	45.83	1.06	−190.00	0.00	30.65
	Beat App	30.00	0.69	−130.00	60.00	41.40
MTB2 4.34 km	Garmin 945	10.56	0.24	−20.00	30.00	13.69
	Polar Ignite	37.22	0.86	−110.00	110.00	42.95
	Beat App	40.56	0.93	−240.00	160.00	65.71
Speed bike 31.5 km	Garmin 945	15.83	0.05	−100.00	20.00	22.74
	Polar Ignite	85.56	0.27	−40.00	180.00	45.43
	Beat App	98.33	0.31	−90.00	260.00	69.02

MAE = mean absolute error (in meter), MAPE = mean absolute percentage error, min = minimum, max = maximum, SD = standard deviation, MTB = Mountain bike, m = meter, km = kilometer.

## Data Availability

The data is not yet publicly available.
